# Effect of Peer Assisted Learning (PAL) education on knowledge, attitude and behavior related to prevention and control of diabetes

**DOI:** 10.1186/s13104-019-4261-9

**Published:** 2019-04-15

**Authors:** Mohammad Taheri, Mitra Amini, Somayeh Delavari, Leila Bazrafkan, Jahanafrooz MazidiMoradi

**Affiliations:** 1 Clinical Education Research Center, Shiraz University of Medical Sciences, Shiraz, Iran; 2 Center for Educational Research in Medical Sciences (CERMS), School of Medicine, Iran University of Medical Sciences, Tehran, Iran; 3 Department of Medical Education, School of Medicine, Iran University of Medical Sciences, Tehran, Iran; 4 Neyriz Health and Care Center, Shiraz University of Medical Sciences, Shiraz, Iran

**Keywords:** Diabetes, Teaching methods, Peer Assisted Learning, Routine lecture, Knowledge, Attitude, Behavior

## Abstract

**Objectives:**

The present study aimed to compare the effect of Peer Assisted Learning (PAL) method and routine lecture on knowledge, attitudes, and behaviors of participants in the prevention of diabetes.

**Results:**

The results showed that one month after the intervention and the implementation of the educational program, the mean scores of the two groups in terms of knowledge, attitude, and behavior increased significantly.

**Electronic supplementary material:**

The online version of this article (10.1186/s13104-019-4261-9) contains supplementary material, which is available to authorized users.

## Introduction

Appropriate knowledge plays an important role in diabetes prevention, attitudes, and behaviors. Individuals can gain this knowledge through a variety of teaching and learning methods, including traditional and non-traditional methods. Lecturing, a traditional method, offers a cheap, fast, and simple way to present knowledge to a lot of learners [1]. As a result, the routine lecture is an effective method for improving the knowledge of large groups [2]. Another, less-traditional method, for improving knowledge of large groups is Peer Assisted Learning (PAL). In this method people from the same group teach each other. PAL is used in a variety of settings and has been found to be particularly useful for programs that focus on preventive activities and promotion of healthy lifestyles [3]. This is because it focuses on peer and patient membership in a group, thus it strengthens the sense of empathy and social identity [4]. PAL creates a learning environment that makes it more comfortable for patients to learn information from their peers and to share their concerns with them [5]. Therefore, PAL is used in diverse settings throughout the world and across different age groups to target a broad range of physical health outcomes [6]. PAL has been used to reduce demands on instructors and improve the overall learning of the trainees [7].

To learn about the effectiveness of PAL as a method for teaching patients about diabetes, we conducted a study aimed at comparing the effect of PAL method to the effect of routine lecture on knowledge, attitudes, and behaviors of employees about diabetes.

## Main text

### Method

We conducted a quasi-experimental study (pretest study + intervention + post-test) with two groups of learners: a PAL group and a control (routine lecture) group.

#### Participants

Sampling was done purposefully using the cluster sampling method. Statistical society was include of 760 people. With an estimation error of one score and standard deviation of 3 and a confidence level of 95%, the sample size was determined to be 35 people in each group (the routine lecture group and the PAL group). Considering the effect of the design as 1.5 times and the loss of 10%, we determined the sample size in each group to be 60 persons.

#### Educational intervention

Educational intervention in the present study was carried out in three stages. The first stage was conducted by selecting a mentor's co-educator and training them, and the second stage was performed by intervention in the peer group. Therefore, first 5 participants from the PAL group were selected as peers for teaching others in the PAL group and these 5 participants were trained for 2 days by an expert teacher about diabetes. After these participants then trained their peers (third stage). In the routine lecture group, another expert teacher participated in a similar training program by using lecture, questions and answers and group discussion about the topics related to knowledge, attitude and diabetes prevention behaviors. After the completion of the training sessions, both groups were given the training booklets.

The content of education in both groups consists of information about the anatomy and pathophysiology of diabetes, common signs and symptoms of diabetes, risk factors of diabetes, organs affected, personal precautions, diabetic foot care, warning signs of hypoglycemia, checking blood sugar at routine intervals, diet plan, physical exercise, chronic complications, obesity and diabetes, self-care and quality of life.

#### Data gathering tools

Data were collected through a researcher-made pretest and posttest consisting of questions about gender, age; questions related to diabetes diagnosis; questions about the attitude about the disease; and questions on the behavioral section including actions such as determining the status of physical activity, weight measurements, blood lipid measurements, blood pressure measurements, healthy eating, cigarette smoking, and blood glucose measurements. A sample of the questionnaire is in Additional file 1. To determine the validity of the content, we sent the questionnaire to 10 faculty members specialized in this field, and after receiving their suggestions, necessary revisions were made in the questionnaire. So, to determine the content validity, the questionnaire was assessed by ten faculty members. Then, using the CVR method, content validity was calculated, which was more than 66 for all questions [8]. In order to determine the reliability of the questionnaire, we used test–retest within a week’s interval. The reliability coefficient of the questionnaire was evaluated using Pearson's correlation coefficient was 0.8.

#### Statistical analysis

Independent t-test, Paired t-test and Chi-square tests were used to compare the responses. SPSS version 24 was used and the significance level of < 0.05 was considered significant for all tests. To control the confounding factors, we performed the random allocation of the groups and the two groups were homogeneous according to age, education, sex, age, marital status; the Kolmogorov–Smirnov test revealed that the distribution of data was normal.

### Results

All of the participants remained in the study until the end of the study, and the questionnaires were answered by all, so the completion rate was %100. The average ages of partitipants in the PAL group and routine lecture group were 37.08 ± 6.08 and 36.27 ± 6.26 respectively. The mean score of knowledge (PAL = 4.38 ± 1.27, routine lecture = 4.36 ± 1.68, p = 0.951), attitude (PAL = 2.06 ± 1.35, routine lecture = 2.08 ± 1.41, p = 0.948), and behavior (PAL = 5.04 ± 2.41, routine lecture = 5.08 ± 1.45, p = 0.921), in both PAL and routine lecture groups before the intervention and education based on the independent t-test was not significantly different (p > 0.05).

One month after the intervention and education, the mean scores of knowledge, attitude, and behavior were significantly increased in both PAL and routine lecture groups (p < 0.05) (Table 1).

**Table 1:** Knowledge, attitude and behavior in both groups before and after the intervention

	PAL				Routine lecture			
	Mean	Standard deviation	Mean difference	p value*	Mean	Standard deviation	Mean difference	p value*
Awareness								
Before intervention	4.38	1.27	15.78	< 0.0001	4.36	1.68	15.79	< 0.0001
After intervention	20.16	0.90			20.15	87		
Attitude								
Before intervention	2.06	1.35	6.64	< 0.0001	2.08	1.41	6.63	< 0.0001
After intervention	8.7	1.07			8.71	1.1		
Behavior								
Before intervention	5.04	2.41	5.04	< 0.0001	5.08	2.45	5.02	< 0.0001
After intervention	10.08	2.11			10.1	1.60		

* Paired t-test

The diabetes preventive behavior in this study included physical activity, weight control, blood pressure measurement, blood lipid profile, diabetes risk assessment, increased healthful food intake (fruit, vegetable, legumes, fish, wholegrain bread, liquid frying oil, limited consumption of out-of-home prepared foods and fried foods), and the use of no cigarettes and any tobacco. In both groups, diabetes prevention behaviors, except for smoking cessation, were significantly different in pretest and posttest (p < 0.05). Smokers needed a longer period of time to stop smoking (Table 2).

**Table 2:** Comparison of the distribution of frequency of the behavior in the two groups before and after the intervention

	PAL					Routine lecture				
	Before intervention		After intervention		p value	Before intervention		After intervention		p value
	Frequency	Percentage	Frequency	Percentage		Frequency	Percentage	Frequency	Percentage	
Having physical activity	14	23.3	44	73.3	< 0.001	13	21.7	45	75	< 0.001
Weight measurement	21	35	56	93.3	< 0.001	22	36.7	55	91.7	< 0.001
Performing a blood lipid test	14	23.3	49	81.7	< 0.001	16	26.7	50	83.3	< 0.001
Blood pressure measurement	21	35	57	95	< 0.001	23	38.3	56	93.3	< 0.001
Consumption of 2 units of fruit per day	11	18.4	40	66.7	< 0.001	13	21.7	42	70	< 0.001
Consumption of 2 units of vegetable per day	15	25	41	68.3	< 0.001	14	23.3	43	71.7	< 0.001
Consumption of 3 servings of legumes per week	24	40	45	75	< 0.001	22	36.6	47	78.3	< 0.001
Consumption of 2 servings of fish per week	13	21.7	25	41.7	< 0.001	10	16.7	26	43.3	< 0.001
Consumption of out-of-home prepared food less than three times a week	32	53.3	37	61.7	< 0.031	34	56.7	38	63.3	0.035
Consumption of whole bread	12	20	29	48.3	< 0.001	14	23.3	30	50	< 0.001
Consumption of liquid frying oil	29	48.3	37	61.7	< 0.008	27	45	36	60	< 0.008
Consumption of fried foods less than two times a week	21	35	33	55	< 0.001	23	38.3	31	51.7	0.021
No cigarettes and any tobacco	58	96.7	58	96.7	1	57	95	57	95	1
Determining the risk of diabetes	13	21.7	51	85	< 0.001	15	25	52	86.7	< 0.001

Also, independent t-test results showed that the difference in the mean score of knowledge (PAL = 15.78 ± 1.32, routine lecture = 15.79 ± 1.79, p = 0.972), attitude (PAL = 6.64 ± 1.59, routine lecture = 6.63 ± 1.72, p = 0.973), and behavior (PAL = 5.04 ± 1.88, routine lecture = 5.02 ± 2.07, p = 0.955) of diabetes in the PAL group after the intervention was not significantly increased compared to that in the routine lecture group (p > 0.05).

### Discussion

Living with a chronic illness can be challenging for a variety of reasons. In order to manage their own illness and take responsibility for their own health, people need knowledge and skills [9]. The benefits of diabetes education has largely been accepted in diabetes care [10]. Recent studies showed that a structured patient therapeutic education may decrease both mortality and the development of diabetes complications [11]. There is some evidence to suggest that interventions including blood glucose awareness training and cognitive behavioral therapy can reduce levels of fear and improve disease management [12]. We wanted to determine the effectiveness of different approaches to providing education on diabetes, so we compared the effect of routine lecture and PAL on promoting knowledge, attitude and behavior related to prevention and control of diabetes in employees.

The results of this study showed that the mean scores of knowledge, attitude, and behavior in both PAL and routine lecture groups increased significantly after the educational intervention. this showed that both PAL and routine lecture had a positive effect in improving the level of awareness of the participants; Bloomgarden et al. study results confirmed this same result [13]. In both the PAL education and routine lecture group, the mean of attitude score also increased significantly.

After the intervention, using group discussion in both groups of diabetes prevention behaviors increased behaviors such as physical activity, weight loss, blood pressure measurements, blood lipid determination, determination of the risk of diabetes, using healthy diet, but it had no effect on quitting smoking; cigarette smokers needed a longer period of time to stop smoking. PAL and the routine lecture groups created appropriate behaviors. This reflects the impact of the educational program and interventions on behavior change and the development of appropriate diabetes prevention behaviors in both groups.

Zhong et al., in their educational intervention using peers and community health personnel, found that interventions by peers and health personnel increased the subjects’ self-efficacy and knowledge, and improved systolic blood pressure and body mass index in diabetes patients [14]. Also, the study carried out by Kargar et al., on the impact of training on osteoporosis prevention by peers and health personnel, showed that training by peers and health personnel was equally effective [15]. The results of other similar studies in medical education, such as Weyrich et al. [16] is consistent with the findings of this study. The results of Abu Moghli et al.’s study did not show any significant difference between the two case and control groups despite the improvement of knowledge and attitude level regarding the physical activity in the performance of the study subjects [17].

The strength of this study was the determination of peer education in preventing diabetes in people who have not yet been diagnosed with diabetes; given the increasing prevalence of diabetes and other non-communicable diseases, we can, with the help of our peers, learn how to choose a healthy lifestyle in the community and reduce the incidence of diabetes and other non-diabetic diseases.

According to the findings of this study, the use of PAL can be considered as effective as routine lecture teaching people about diabetes prevention and control. In addition, peers can, along with other health care providers, help to promote knowledge, attitude, and behavior within the community on diabetes and other diseases. The results of our study, combined with the results of prior studies, indicate that peer groups can be used as an effective and low-cost method for providing education, and an acceptable alternative to education provided by health care personnel and helath educators.

## Limitation

The limitation was that we could not actually observe participants.

## Additional file

**Additional file 1.** Diabetes Questionnarie.

## Abbreviation

PAL: Peer-Assisted Learning
