# Supplementary material for: Effect of Peer Assisted Learning (PAL) education on knowledge, attitude and behavior related to prevention and control of diabetes
Source: BMC Res Notes. 2019 Apr 15;12:227. doi: 10.1186/s13104-019-4261-9 (PMC6466761; doi:10.1186/s13104-019-4261-9)
Supplement: Supplementary file 1 — Additional file 1. Diabetes Questionnarie. [file 13104_2019_4261_MOESM1_ESM.docx]

**Assessment of knowledge, attitude, practice about diabetes**

**Questionnaire code**:

1. before intervention b) after intervention

**Educational method:** a) Lecture b) Peer Assisted Learning

**Sex** a) male b) female

**Age …** years

**Marital status:** a) single b) married c) divorced/separated

**Academic degree:** a) Illiterate b) Reading and writing literacy c) Guidance school diploma d) High school diploma e) Associate’s degree f) Bachelor’s degree and higher educations

**Hypertension history:**  a) yes b) no

**Awareness questions**

Please read these questions carefully and mark the correct answer.

1) What kind of disease is diabetes?

a) Infectious diseases

b) Non-infectious diseases

c) Psychiatric diseases

d) Anemia

2) What is the symptoms of diabetes?

a) polydipsia- polyphagia- dyspnea

b) polyuria- polyphagia- nausea

c) polydipsia- polyphagia-polyuria

d) sever pain- polyuria- dyspnea

3) Which way is the most important way to prevent diabetes?

a) Weight loss-vegetables consumption-quit smoking

b) Exercise-enough eating-control of stress

c) Control of stress-weight loss-exercise

d) Weight loss-exercise-vegetables consumption

4) Which statement is a sign of severe blood sugar drop in diabetic patients?

a) vertigo- palpitation- sweating- speech problem

b) vertigo- palpitation- speech problem- polydipsia

c) polyuria- palpitation- sweating- speech problem

d) vertigo- sweating- nausea and vomiting- weight loss

5) Which choice shows the main reason of blood sugar drop in diabetic patients?

a) Not enough food consumption-excessive exercise and activity

b) Severe mental stresses-excessive exercise and activity

c) Physical illness such as infection and fever-surgery operation

d) Excessive exercise and activity- physical illness such as infection and fever

6) What is the best way to diagnose diabetes?

a) Physical examination by physician

b) Urine analysis test

c) Blood sugar test

d) Platelet test

7) Which choice is the complication of diabetes?

a) Eye problem-vascular and neurological complications (diabetic foot) -skin lesion-liver toxicity

b) Liver toxicity- cardiac problem- renal problem- skin lesion

c) Vascular and neurological complications (diabetic foot)-eye problem-liver toxicity-renal problem

d) Eye problem-vascular and neurological complications (diabetic foot)- cardiac & renal problem

8) Which one is not the most important factor in the appearance of diabetes II?

a) Old age

b) Obesity and lack of exercise

c) History of diabetes in pregnancy

d) Disorder of immune system

9) Which of the following nutriments have less sugar?

a) Sangak bread, lentil and pinto bean

b) Lavash bread, rice and peas

c) Mashed potato, carrot and machine Lavash bread

d) Mung bean, potato and green fava been

10) Who are the individuals with the most symptoms of diabetes II, respectively?

a) Obese people- children

b) Adult – teenager

c) Obese people - adult

d) Adults - teenager

11) Which of the following procedure is recommended in diabetic patients with blood sugar drop (hypoglycemia)?

a) Sugar and water consumption

b) Insulin injection

c) Half glass juice

d) a & c

12) Which choice is the most environmental factor related to the appearance of diabetes II?

a) Genetics

b) Obesity

c) Lack of exercise

d) Mental stresses

13) What kinds of diabetes symptoms are seen in ulcer formation and diabetic foot condition, respectively?

a) Numbness-disorder of immune system

b) Nervous problems-vascular and blood supply disorder

c) Virus - disorder of immune system

d) Virus - vascular and blood supply disorder

14) Which statement expresses the normal fasting blood sugar level properly?

a) Less than 70 milligram per deciliter (mg/dl)

b) 70-99 milligram per deciliter

c) 100-125 milligram per deciliter

d) Higher than 126 milligram per deciliter

15) Which one is necessary to increase in the diabetic people’s food program?

a) Non-sweet fruits and vegetables

b) Starch nutrition

c) Legume

d) a & c

16) How much does weight loss need to decrease the primary weight in obese persons with diabetes?

a) 10% of the patient weight

b) 15% of the patient weight

c) 20% of the patient weight

d) 25% of the patient weight

17) What is the optimum level of hemoglobin A1C in diabetic patients?

1. less than 5%
2. less than 6%
3. less than 7%
4. less than 8%

18) In what periods diabetic patients have to do periodic eye examination?

1. every 6 months
2. every 3 months
3. every year
4. every 2 year

19) On what intervals a 24-hour urine test is done in diabetic patients?

a) It is done at least once a year after 5 years of the appearance of diabetes I

b) It is done at least once every 6 months in diabetic patients’

c) It is done from the onset of the diagnosis of diabetes II

d) a and c

20) Which exercise has the most effect related to preventing and controlling of diabetes?

a) Easy and regular exercise at least 3 times per week

b) Moderate and regular exercise at least 3 times per week

c) Easy and regular exercise at least 5 times per week

d) Moderate and regular exercise at least 5 times per week

21) Which choice is the optimum control of blood sugar level in diabetic patients?

1. Fasting blood sugar 70-130 mg/dl- 2 hours blood sugar less than 180 mg/dl
2. Fasting blood sugar 60-145 mg/dl- 2 hours blood sugar less than 185 mg/dl
3. Fasting blood sugar 60-147 mg/dl- 2 hours blood sugar less than 187 mg/dl
4. Fasting blood sugar 70-135 mg/dl- 2 hours blood sugar less than 175 mg/dl

**Attitude questions**

Please study these sentences carefully and mark the part which expresses your idea.

|  |  | I agree | I have no idea | I disagree |
| --- | --- | --- | --- | --- |
| 1 | Diabetes is a chronic and lifelong disease. |  |  |  |
| 2 | Due to high blood sugar, a diabetes patient cannot use sweet drinks or some sugars. |  |  |  |
| 3 | The best time for exercise is 1 to 3 hours after eating. |  |  |  |
| 4 | Endurance and heavy exercises (weightlifting) is suitable for diabetic people. |  |  |  |
| 5 | Diabetes is the most common cause of leg amputation, blindness, chronic renal failure and cardiovascular complications in the world. |  |  |  |
| 6 | Cigarettes consumption increases the effects of diabetes. |  |  |  |
| 7 | It is necessary for diabetic patients to visit medical doctor regularly. |  |  |  |
| 8 | A diabetic patient must exercise regularly. |  |  |  |
| 9 | It is important to follow the scheduled diet to control diabetes. |  |  |  |
| 10 | Diabetes will challenge human life. |  |  |  |

**Behavioral questions**

Please study these sentences carefully and mark the part which indicates your behavior.

|  | **Body activity** | | | | | | | |
| --- | --- | --- | --- | --- | --- | --- | --- | --- |
|  |  | Do you have regular exercise schedule (at least 5 days per week)?  Yes  No | | | | | | |
|  |  | If yes, how many days do you have regular body activity?  Less than 5 days per week  5 days and more per week | | | | | | |
|  |  | How long do you usually exercise daily?  Less than 30 minutes per day  30 minutes and more per day | | | | | | |
|  |  | **Determine the severity of your body activity:**  **1)** **Easy activity** (common activities such as housekeeping, washing machine, irregular walking (walking to buy or walking to the workplace), work in the house garden, sweeping and so on)  **2) Moderate body activity** (such as fast walking, fun and slow cycling, swimming, badminton, fun rafting, carrying light burdens, frequent short stair steps)  **3) Heavy body activity** (such as running (track and field), weightlifting, martial sports, basketball, volleyball, mountain climbing, rapid cycling, ski, tennis and rapid rafting, carrying heavy burdens and construction and excavation work) | | | | | | |
|  | **Weight** |  | | | | | | |
|  |  | Have you measured your weight during last year? | | |  | |  | |
|  |  | If yes, have your doctor said that you have overweight and obesity? | | |  | |  | |
|  |  | If yes, do you have a schedule to decrease overweight and obesity? | | |  | |  | |
|  | **Blood lipid** |  | | | | | | |
|  |  | Have you ever done blood lipid test (cholesterol, TG, LDL, HDL)? | | |  | |  | |
|  |  | If yes, have your doctor said that you have a high blood lipid? | | |  | |  | |
|  |  | If yes, If yes, do you have a schedule consisting of using drug, exercise and proper nutriment to control it? | | |  | |  | |
|  | **Blood pressure** |  | | | | | | |
|  |  | Have you measured your blood pressure during last year? | | |  | |  | |
|  |  | If yes, have your doctor said that you have high blood pressure? | | |  | |  | |
|  |  | If yes, do you have a schedule consisting of using drug, exercise and proper nutriment to control it? | | |  | |  | |
|  | **Nutrition** |  | | | | | | |
|  |  | Do you consume at least 2 fruit units per day?  (One fruit unit consists of a moderate fruit (Orange, sweet lemon, apple, kiwis or half of banana), half glass of small fruits such as grapes, pomegranate, sour cherry or quarter cantaloupe or one-eighth of a moderate watermelon.) | | |  | |  | |
|  |  | Do you consume at least 2 vegetable units per day?  (Vegetables such as green leafy vegetables, types of cabbages, green bean, zucchini, peas and green fava bean which one unit of them consists of a glass of raw vegetables or a half glass of cooked vegetables.) | | |  | |  | |
|  |  | Do you consume legume at least 3 times a week?  (One unit of legume is equal to half of glass of raw legume or a glass of cooked legume consists of chickpea, lentil, bean, cotyledon and mung bean.) | | |  | |  | |
|  |  | Do you consume fresh fish and the other seafood like shrimp and so on? | | |  | |  | |
|  |  | Do you consume fast food less than 3 times per week? | | |  | |  | |
|  |  | Do you consume bran bread? | | |  | |  | |
|  |  | Do you consume liquid oil? | | |  | |  | |
|  |  | Do you consume fried food less than 2  times per week? | | |  | |  | |
|  | **Cigarettes and smoking** |  | | | | | | |
|  |  | Do you use cigarettes or any type of smoking? | | | |  | |  |
|  | If yes, which one do you use? 1) cigarettes  2) hookah  3) tobacco pipe and chibouk  4) The other smoking with their names… | | | | | | | |
|  |  | | Do you have a plan to quit it? |  | | |  | |
|  | **Blood sugar test** | |  | | | | | |
|  |  | | Have you visited health workers and doctors to determine the risk situation? |  | | |  | |
|  |  | | If yes, are you at risk for diabetes? |  | | |  | |
|  |  | | If yes, have you done fasting blood sugar test? |  | | |  | |
|  |  | | If yes, was your fasting blood sugar test normal? |  | | |  | |

Date:
